# Microscopic Examination of the Tartar of the Teeth

**Published:** 1844-03-23

**Authors:** 


MICROSCOPIC EXAMINATION OF THE TARTAR OF THE TEETH.

Hitherto this calcareous incrustation has always been regarded as a mere inorganic deposit from the saliva; but M. Mandl has been led by his recent observations to adopt a very different opinion respecting it. According to this gentleman, the tartar of the teeth is almost entirely composed of the skeletons or cases of infusory animalculæ, agglutinated together by dried mucus; just as certain soils (so says the great German microscopic naturalist, Ehrenberg,) consist of fossil infusoria.

If a portion of the mucosity, which is adherent to the teeth, be mixed with a little water and heated, and then subjected to microscopic inspection, it will be found to exhibit a number of infusory animalculæ, which are in active motion. Their shape is identical with that of the animalculæ known by the name of Vibrio. The presence of living infusoria in mucosities was known to Leuwenhock; but the fact seems to have been forgotten, until revived by M. Mandl. Having satisfied himself that they exist in the mucosities of the mouth, he set himself to ascertain whether they contributed to the formation of the tartar of the teeth; and he was not long of discovering that this substance chiefly, if not entirely, consists of dead vibrios cemented together by dried mucus. From this circumstance, may we not infer that these animalculæ are provided with an inorganic calcareous covering to their bodies?

It may be useful here to state, that, according to the analysis of M. Vauquelin, the tartar of the teeth consists, in the 100 parts, of 66 of phosphate of lime, 9 of the carbonate, 14 of animal matter, and 3 of oxide of iron and phosphate of magnesia.—*Medico-Chirurgical Review.*

